# An Influenza A/H1N1/2009 Hemagglutinin Vaccine Produced in *Escherichia coli*

**DOI:** 10.1371/journal.pone.0011694

**Published:** 2010-07-22

**Authors:** José M. Aguilar-Yáñez, Roberto Portillo-Lara, Gonzalo I. Mendoza-Ochoa, Sergio A. García-Echauri, Felipe López-Pacheco, David Bulnes-Abundis, Johari Salgado-Gallegos, Itzel M. Lara-Mayorga, Yenny Webb-Vargas, Felipe O. León-Angel, Ramón E. Rivero-Aranda, Yuriana Oropeza-Almazán, Guillermo M. Ruiz-Palacios, Manuel I. Zertuche-Guerra, Rebecca M. DuBois, Stephen W. White, Stacey Schultz-Cherry, Charles J. Russell, Mario M. Alvarez

**Affiliations:** 1 Centro de Biotecnología-FEMSA, Tecnológico de Monterrey at Monterrey, Monterrey, México; 2 Department of Structural Biology, St. Jude Children's Research Hospital, Memphis, Tennessee, United States of America; 3 Department of Infectious Diseases, St. Jude Children's Research Hospital, Memphis, Tennessee, United States of America; Johns Hopkins School of Medicine, United States of America

## Abstract

**Background:**

The A/H1N1/2009 influenza pandemic made evident the need for faster and higher-yield methods for the production of influenza vaccines. Platforms based on virus culture in mammalian or insect cells are currently under investigation. Alternatively, expression of fragments of the hemagglutinin (HA) protein in prokaryotic systems can potentially be the most efficacious strategy for the manufacture of large quantities of influenza vaccine in a short period of time. Despite experimental evidence on the immunogenic potential of HA protein constructs expressed in bacteria, it is still generally accepted that glycosylation should be a requirement for vaccine efficacy.

**Methodology/Principal Findings:**

We expressed the globular HA receptor binding domain, referred to here as HA_63–286_-RBD, of the influenza A/H1N1/2009 virus in *Escherichia coli* using a simple, robust and scalable process. The recombinant protein was refolded and purified from the insoluble fraction of the cellular lysate as a single species. Recombinant HA_63–286_-RBD appears to be properly folded, as shown by analytical ultracentrifugation and bio-recognition assays. It binds specifically to serum antibodies from influenza A/H1N1/2009 patients and was found to be immunogenic, to be capable of triggering the production of neutralizing antibodies, and to have protective activity in the ferret model.

**Conclusions/Significance:**

Projections based on our production/purification data indicate that this strategy could yield up to half a billion doses of vaccine per month in a medium-scale pharmaceutical production facility equipped for bacterial culture. Also, our findings demonstrate that glycosylation is not a mandatory requirement for influenza vaccine efficacy.

## Introduction

The emergence of pandemic H1N1 subtype influenza in April 2009 emphasizes the need for rapid methods to manufacture large quantities of influenza vaccine. To curtail a second wave of influenza A/H1N1/2009 in the U.S.A, it was estimated that up to 70% of citizens would need to be vaccinated by the Fall of 2009 [1]. More than 20% vaccination coverage has been proposed based on other reports [2]. While 20% vaccine coverage was at least partially achieved in some First World European countries, in nations such as México (the epidemiological epicenter of the current pandemic), sufficient vaccine dosages were not available even by March 2010.

All commercial influenza vaccines are produced by propagating the virus in embryonated chicken eggs. Further processing is then needed to separate and inactivate viral particles and to purify the hemagglutinin (HA) protein, the primary vaccine antigen. This technology is slow and requires one embryonated egg per vaccine dose [3]. To vaccinate one third of the population in the United States and México, 150 million eggs would be required, and an additional 150 million doses would be needed for the rest of Latin America.

Several alternative strategies have been proposed to produce pandemic and seasonal influenza vaccines [4], [5]. These include viral culture in mammalian cells [5]–[7] and the use of recombinant proteins [3], [8]–[12]. The concept of producing subunit influenza vaccines was first proposed three decades ago [13]. The expression and purification of a single antigenic protein in bacterial culture [3], [10], [11] may be the simplest and fastest strategy for generating large quantities of new influenza vaccines. In fact, the development of a bacterial clone capable of producing an antigen against a new influenza strain would require less than one week, and scaling up production using bioreactors would allow the generation of hundreds of thousands of doses in less than a day. Moreover, recombinant vaccines produced in bacteria, free of other viral and cellular components, are expected to reduce complications associated with whole virus vaccines such as pyogenic reaction and Guillain-Barre syndrome [13].

One concern is that complete viral particles may be orders of magnitude more immunogenic than recombinant peptides [11] because the former are polyantigenic and undergo post-transcriptional modifications such as glycosylation. Commercial vaccines based on recombinant technology are presented as “virus like particles” and/or are expressed in eukaryotic systems capable of glycosylation. For example, GARDASIL® (Merck) against Human Papilloma virus, and Recombivax® (Merck) against Hepatitis B virus are expressed in *Saccharomyces cerevisiae*. Nonetheless, multiple and single antigen experimental vaccines produced in bacteria have proved to be protective in animal models [14], [15]. In the case of influenza viruses, there is experimental evidence to suggest that HA glycosylation might be important for proper folding [16] and virus-host receptor recognition [5], [6], [17]–[19], but not for immunogenicity to any significant degree [3], [10], [11], [19]–[21].

## Results

### Design and expression of the HA receptor-binding domain

**Figure 1:**
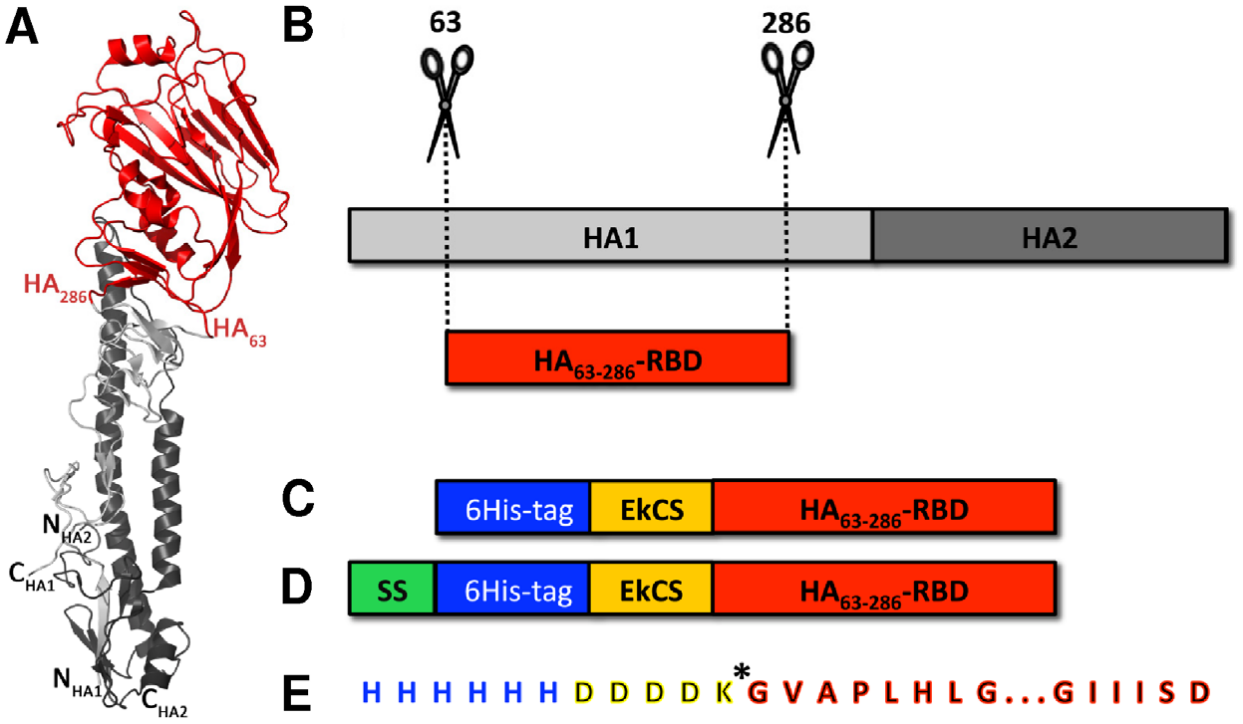
Construction of HA_63–286_-RBD. (A) Crystal structure of HA protein (PDB entry 1RUY) showing HA1 (light grey), HA2 (dark grey), and the globular domain of HA_63–286_-RBD (red) used for these studies. (B) Schematic for the construction of HA_63–286_-RBD. The cDNA sequence encoding residues 63–286 of influenza A H1N1 virus (without transmembrane regions) was cloned for expression in *Escherichia coli*. (C) Schematic representation of the HA_63–286_-RBD containing an N-terminal 6×Histidine tag and an enterokinase cleavage sequence (EkCS). (D) Same as (C) except that this construct contains a periplasmic signal sequence. (E) Amino acid sequence of the N-terminus in both (C) and (D). * indicates the enterokinase cleavage site.

In this paper, we document the production of a recombinant HA receptor-binding domain (HA RBD) in *E. coli* that specifically binds serum antibodies from positive influenza A H1N1/2009 patients. When intramuscularly administered, the protein triggers a specific immune response, produces neutralizing antibodies, and provides protection against influenza A/H1N1/2009 challenge in ferrets. This 25 kDa protein comprises a highly conserved region of the HA1 domain of the hemagglutinin of the A/H1N1/2009 virus spanning amino acids 63 to 286 of the native sequence, and is therefore designated HA_63–286_-RBD (residues 55–271 in H3 numbering, v.gr. Accession No. ACQ99608) (Figure 1a, 1b). In addition, it contains all of the predicted antigenic sites for the HA protein of the A/H1N1/2009 strain [22]. A sequence encoding a six-histidine purification tag was added at the N-terminus of the protein, and an enterokinase cleavage site (EKCS) was added to facilitate tag removal (Figure 1c, 1d, 1e).

**Figure 2:**
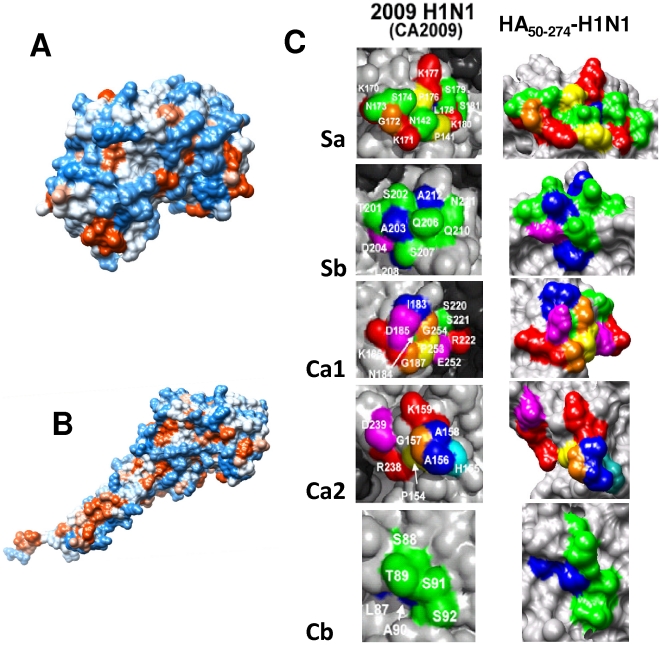
Surface hydrophobicity and antigenic structure of protein HA_63–286_-RBD. (A) Hydrophobic (red) and hydrophilic regions (blue) at the surface of protein HA_63–286_-RBD calculated by simulations; (B) The hydrophobicity map of the HA1 subunit expressed by Chiu *et al* (10) is presented for comparison. (C) Simulation results show that protein HA_63–286_-RBD preserves the conformational antigenic sites Sa, Sb, Ca1, Ca2, Cb computationally predicted by Igarashi *et al.* [22] for the HA of the influenza A H1N1/CA2009 virus. Three dimensional structures were obtained using Swiss-model. The full length HA of the Influenza A H1N1/1918 virus [23] was taken as a template for the estimation of the most probable structure of protein HA_63–286_-RBD. Visualization and highlighting of immunogenic sites was done using UCSF-Chimera. The structure of the antigenic epitopes of the HA of the influenza A H1N1/CA2009 virus was taken from Igarashi *et al.* [22]. They are also consistent with structural data published recently by Xu *et al* [41].

**Figure 3:**
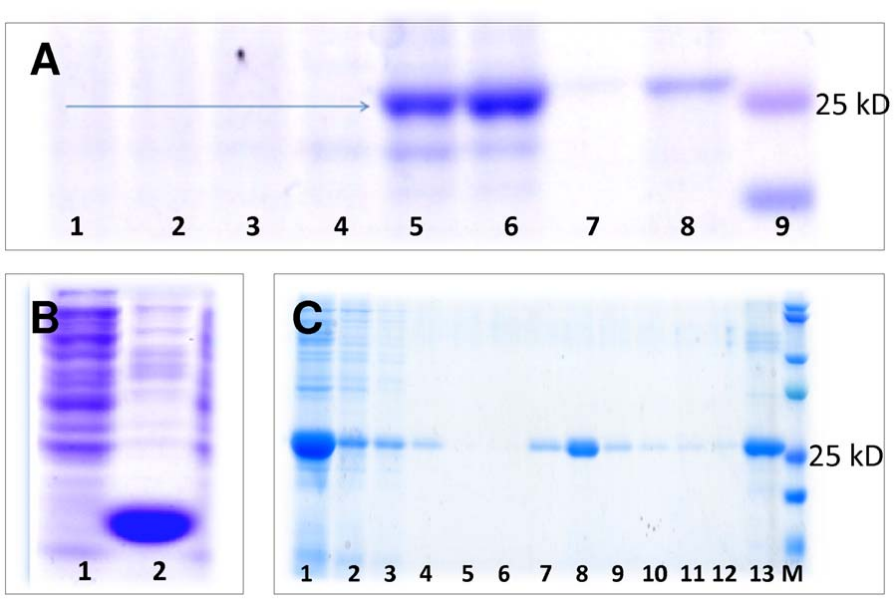
Expression of HA_63–286_-RBD in *E. coli*. (A) Protein profile of cell lysates from culture experiments of *E. coli* C41, BL21 (DE3) pLysS or Rosetta-gami transformed with genes to produce (1) GFP+histidine tag (clone C41 1); (2) GFP+histidine tag (clone C41 2); (3) GFP+histidine tag (clone C41 3); (4) negative control, C41(5) HA_63–286_-RBD (clone C41 1); (6) HA_63–286_-RBD (clone C41 2); (7) HA_63–286_-RBD (clone Rosetta-gami clone 1); (8) HA_63–286_-RBD (clone Rosetta-gami clone 2). (9) Precision Plus Kaleidoscope molecular mass ruler showing 25 kD (pink) and 20 kD (blue) bands. The blue arrow indicates the 26 kD band corresponding to HA_63–286_-RBD. (B) SDS-PAGE showing (1) the soluble and (2) insoluble fraction of the C41 strain lysate after 8 hours induction with 1mM IPTG. (C) SDS-PAGE showing the protein profiles at different stages of recovery, purification and on-column refolding. (1) Crude lysate of the 8M urea solubilized inclusion bodies, (2) Unbound fraction, (3) 1st wash step, (4) 2nd wash step, (5,6) refolding steps, (7–12) Elution fraction using imidazole 300 mM, (13) chromatographic resin. (M) Precision Plus Kaleidoscope molecular mass ruler.

The selection of this precise HA subdomain purposely excludes all residues of the metastable HA2 stalk domain including the hydrophobic fusion peptide and transmembrane domain. Computer simulations predict that the isolated HA1 receptor-binding domain has much less surface hydrophobicity than the entire HA protein ectodomain (compare Figure 2a and 2b) while still preserving its antigenic structure (Figure 2c). For these simulations, the full length HA of the Influenza A H1N1/1918 virus [23] was taken as a template for the estimation of the most probable structure of protein HA_63–286_-RBD. Given the close similitude in primary and tertiary structure between the HA H1N1/2009 and the HA H1N1/1918 protein [22], the accuracy of the predicted structure of the HA_63–286_-RBD is expected to be high. Hydrophobicity minimization is generally conducive to higher expression levels in *E. coli* which typically recognize prominent hydrophobic regions as being misfolded and subsequently degrade them [24]. Indeed, in our experiments, high production levels of the complete H1N1/2009 HA1 subunit were not achievable using either conventional *E. coli* strains or *E. coli* strains BL21 (DE3) pLysS variants C41 and C43 from Lucigen® Corporation (Middleton, WI) which are known to successfully express transmembrane proteins [25], [26]. Protein HA_63–286_-RBD was expressed in both *E. coli* Rosetta-gami and C41, but not in C43. Producer strains were deposited at ATCC® under Patent Deposit Designation PTA-10320. In terms of global yield and growth rate, clones derived from the *E. coli* C41 strain were more suitable for large-scale production (Figure 3a). Specific growth rates of 1.69 h^−1^ and average yields of 3.4 g/L were observed at 5L scale settings after 12 h cultivation.

### Protein recovery, purification and refolding

In these culture conditions, practically all of the recombinant protein was produced as insoluble inclusion bodies (Figure 3b). Although this facilitated the primary recovery of protein, the proper folding and resulting bioactivity of the recombinant protein recovered from inclusion bodies requires an effective method to solubilize, refold and purify the protein [10], [24]. By optimizing a recovery and refolding procedure, we eventually obtained a bioactive protein that recognizes antibodies from serum of H1N1/2009 positive patients and provides protection against virus infection in the ferret model. Briefly, HA_63–286_-RBD was recovered using standard chemical lysis procedures, dissolved in 8M urea, and refolded and purified by Immobilized Metal Affinity Chromatography (IMAC) using 400 mM arginine in PBS at pH 8 (Figure 3c). The refolded protein was eluted using 150 mM imidazole at pH 7. This simple purification scheme produced HA_63–286_-RBD solutions in the range of 400 to 650 mg/L with purities exceeding 99.5%, as estimated by microelectrophoresis using an Experion® platform from Bio-rad (Hercules, CA). At the present time, we observe an overall process yield of ≈0.02 g/L of bioreactor volume. After process optimization, average overall yields of 0.5–1.0 g/L (refolded protein per Liter of bacterial culture) could be expected.

**Figure 4:**
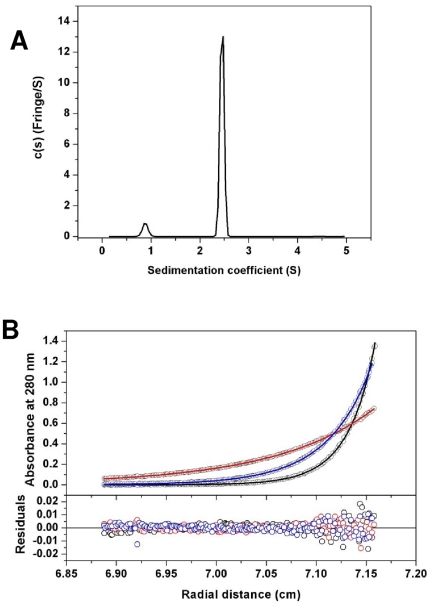
Analytical ultracentrifugation of HA_63–286_-RBD. (A) The sedimentation velocity profiles (fringe displacement) were fitted to a continuous sedimentation coefficient distribution model c(s). The experiment was conducted at a loading protein concentration of 0.45 mg/mL in 10 mM Tris pH 8.0, 100 mM NaCl at 20°C and at a rotor speed of 60,000 rpm. The s-values of the proteins are listed in Table 1. (B) Absorbance scans at 280 nm at equilibrium are plotted *versus* the distance from the axis of rotation. The protein was centrifuged in the above buffer at 4°C for at least 24 h at each rotor speed of 20, 30 and 38 k rpm. The *solid lines* represent the global nonlinear least squares best-fit of all the data sets to a monomer-dimer self-association model with a very weak K_D_ (288 mM). For clarity, only the loading protein concentration of 5 µM is shown. The r.m.s. deviation for this fit was 0.0037 absorbance units.

We further characterized the folded state of protein HA_63–286_-RBD in solution by analytical ultracentrifugation, specifically using sedimentation velocity and equilibrium analysis assays. Both experiments showed that HA-RBD exists mainly as a monomer in solution (Figure 4), and there are no dimmers observed in the c(s) distribution profile (Figure 4a) at the concentration used. The analytical results are presented in Table 1. The frictional ratio value (*f/f_0_* – value) of 1.30 reflects a slightly elongated globular protein, consistent with the predicted three-dimensional structure. The standard s-value, *s^0^_20,w_* (water as solvent at 20°C and zero concentration), and frictional ratio calculated with the standard *s*-value (in parenthesis) are also listed in Table 1. The sedimentation equilibrium data do not fit quite as well to a discrete single monomer species model which predicts a mass value of 28,585 Da, slightly larger than the monomeric molecular weight. The dissociation equilibrium constant of the monomer-dimmer self-association model determined from the equilibrium data is K_D_ = 288 mM, and this suggests a very weak dimerization interaction (root mean square deviation of the model was 0.0037 absorbance units at 280 nm; Figure 4b).

**Table 1:** Summary of results of velocity experiment of HA-RBD in 10 mM Tris pH 8, 100 mM NaCl at 20°C.

Sample^a^	*s_20_* (Svedberg)^b^	*s^0^_20,w_* (Svedberg)^c^	*M* (Da)^d^	*f/f_0_* e
Monomeric HA-RBD (0.45)	2.45 (92%)	2.51	26,700 (26,378)	1.30(1.29)
others (0.03)	0.88 (8%)	N/D	N/D	N/D

*^a^*Concentration of peak in mg/mL in parenthesis.

*^b^*Sedimentation coefficient taken from the ordinate maximum of each peak in the best-fit *c(s)* distribution at 20°C with percentage protein amount in parenthesis. Sedimentation coefficient (*s*-value) is a measure of the size and shape of a protein in a solution with a specific density and viscosity at a specific temperature.

*^c^*Standard sedimentation coefficient (*s^0^_20,w_* -value) at zero concentration, in water at 20°C.

*^d^*Molar mass values taken from the *c(s)* distribution that was transformed to the *c(M)* distribution. The theoretical mass of the monomer is given in parenthesis.

*^e^*Best-fit weight-average frictional ratio values *(f/f_0_)*_w_ taken from the *c(s)* distribution. The frictional ratios calculated with *s^0^_20,w_* -values via the v-bar method (SEDNTERP) is in parenthesis.

### HA_63–286_-RBD specifically recognizes antibodies from H1N1-infected subjects

**Figure 5:**
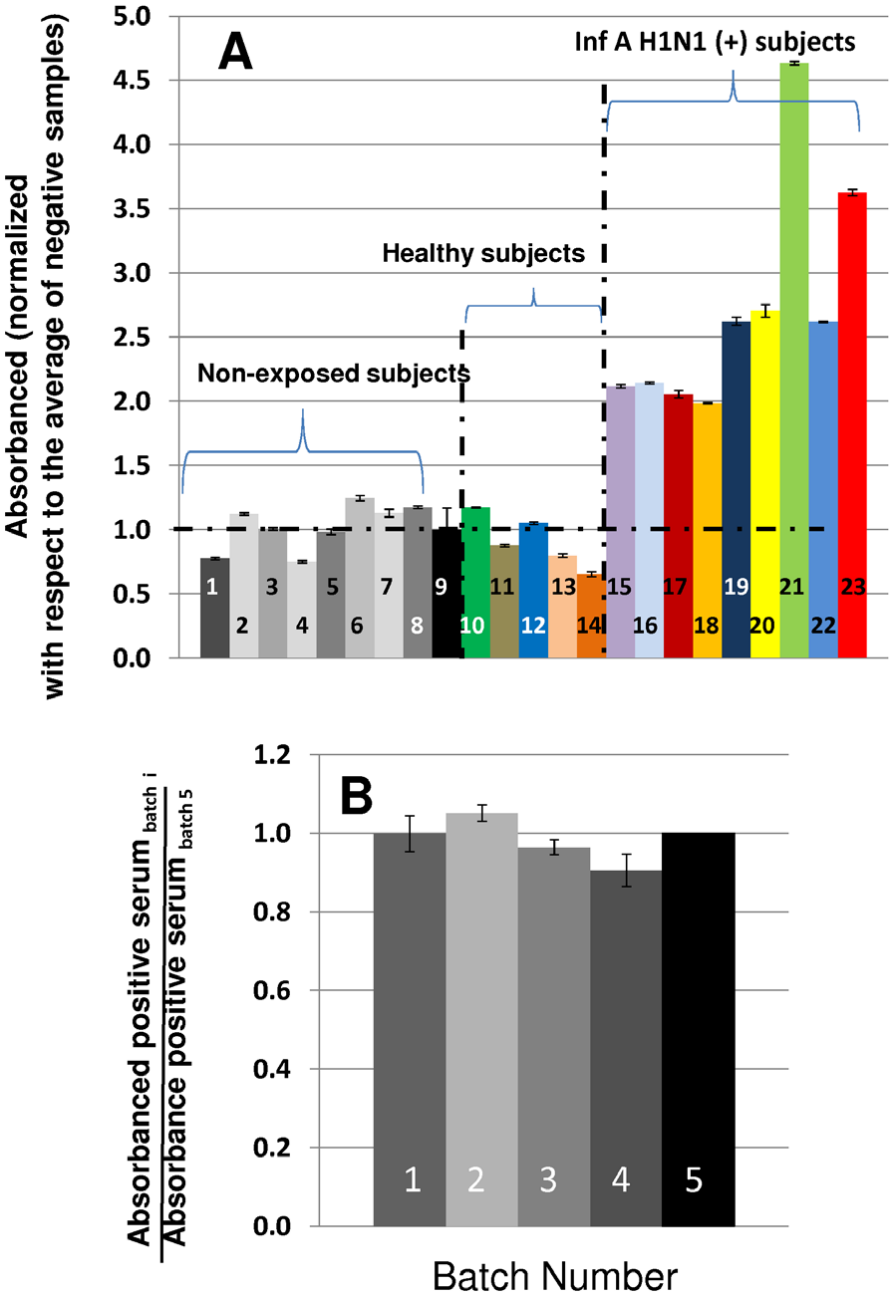
Serum from patients infected with Influenza A H1N1/2009 specifically recognize HA_63–286_-RBD. (A) Bars 1–8, in gray tones, correspond to absorbance signals from non-exposed subjects (samples taken from March to May 2008). Bar 9, in black, shows the average absorbance value of samples 1 to 8. Bars 10 to 14, shown in colour, correspond to absorbance signals from Influenza A/H1N1 negative subjects. Bars 15–23, shown in colour, correspond to absorbance signals from samples of Influenza A H1N1 positive subjects (diagnosed two or three weeks previously by RT-PCR). Error bars represent one standard deviation (B) Proper refolding (biorecognition of antibodies from a positive patient), was evaluated for 4 different production batches of HA_63–286_-RBD. Batch 5 is a reference batch where HA_63–286_-RBD was expressed in its soluble form using a signal peptide for periplasmic expression.

The resulting HA_63–286_-RBD protein is specifically recognized by antibodies in serum samples from patients positive for the 2009 H1N1 virus (Figure 5). In comparative experiments, serum from positive patients diagnosed by the RT-PCR protocols established by the CDC and recommended by the WHO [27], or serum from subjects negative for influenza A H1N1, were measured in an HA_63–286_-RBD-specific ELISA as described by Alvarez *et al.* [28]. At 1∶50 dilution, the absorbance signal observed in samples from positive patients was between 2 to 4-fold higher when compared to signal from samples from negative subjects (Figure 5a).

To establish if HA_63–286_-RBD obtained from inclusion bodies is properly folded, a soluble form of HA_63–286_-RBD was produced by expression in *E. coli* BL21 (DE3) pLysS variant C41 using a genetic construction that included a signal peptide for periplasmic expression [29] and a 6His tag sequence (Figure 1d). Extraction from the periplasmic space was performed using a saline gradient, and practically all of the protein was found in solution, as confirmed by a western blot assay using anti-histidine antibodies. The protein was recovered and purified by affinity chromatography using its histidine tag. Yields of this soluble version of HA_63–286_-RBD are two orders of magnitude lower than its refolded analog, which would make its large scale production unfeasible, but soluble HA_63–286_-RBD is useful as a reference for proper folding.

Selective biorecognition of native soluble and refolded HA_63–286_-RBD by antibodies from the sera of positive influenza A H1N1/2009 subjects was then compared. Refolded HA_63–286_-RBD exhibited more than 90% selective biorecognition with respect to native soluble HA_63–286_-RBD. This was a consistent observation among different batches of product (Figure 5b).

### HA_63–286_-RBD is immunogenic in ferrets

**Figure 6:**
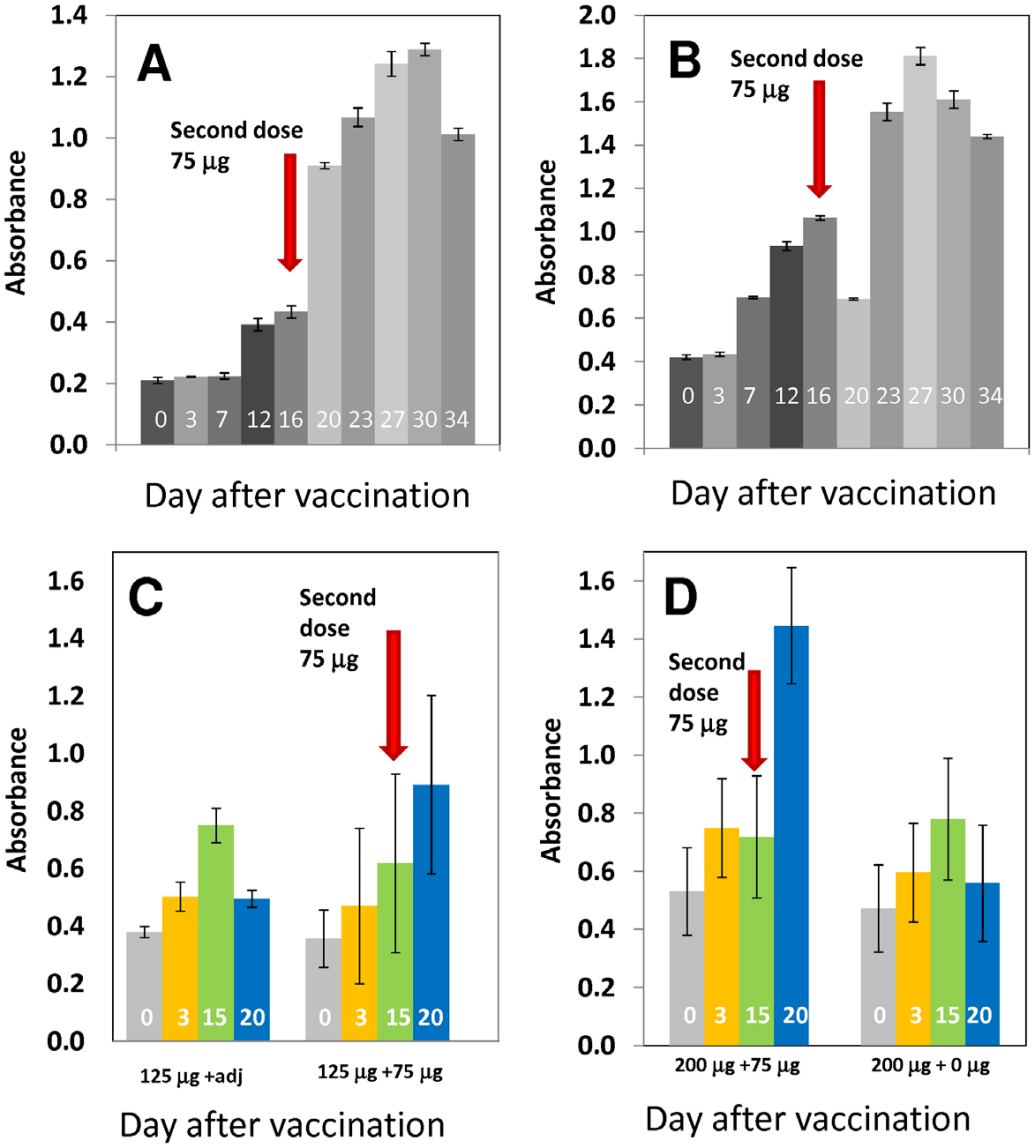
Immunogenic activity of HA_63–286_-RBD. (A) Evolution of specific immune response of ferret 4A to intramuscular application of HA_63–286_-RBD. Arrows indicate the days at which dosages of the protein were administered. (B) Evolution of a specific immune response of ferret 4C to sub-dermal application of HA_63–286_-RBD. Arrows indicate the days at which dosages of the protein were administered. Error bars indicate one standard deviation for three replicates in an ELISA experiment. Specific immune response is expressed in absorbance units (absorbance signal in the assay with ferret serum minus absorbance in negative control). (C and D) Effect of adjuvant on immune response and comparison of immunogenic activity of different doses of HA_63–286_-RBD administered to ferrets. Specific immune response is expressed in absorbance units (absorbance signal in the assay with ferret serum minus absorbance in negative control) at days 0 (gray), 3 (yellow), 15 (green), and 20 (blue) after the first vaccination dose. Error bars indicate one standard deviation with respect to the mean value for replicates of the experiment in different ferrets. Arrows indicate days of application of a second dose (if administered).

The immunogenic and protective potential of HA_63–286_-RBD was evaluated in experiments with ferrets, the preferred animal model for influenza studies [30]–[33]. Sixteen ferrets were intramuscularly administered with different doses of HA_63–286_-RBD with or without adjuvant as described in Table 2. Five additional animals were used as non-vaccinated controls. Serum was collected at different times post-vaccination for 34 days and analyzed for HA-specific antibodies by ELISA. Our results suggest that the immune response to the HA_63–286_-RBD prime alone is variable. Most animals (56.25%) exhibited a moderate primary response that was clearly observable by day 6 or 7 post-vaccination (as in ferret 4C; Figure 5b). Others (43.75%) did not display any significant primary response (as in ferret 4A; Figure 6a). However, all ferrets subjected to a boost at day fifteen exhibited a significant sera IgG response between four and six days after the boost injection. No statistically significant difference was observed between the 125 µg treatments with or without adjuvant (Figure 6a). However, our results suggest that the antibody response is dose dependent. Figures 6c and 6d show the specific antibody levels observed in animals vaccinated with 125 µg and 200 µg dosages. The antibody response was similar at both dosage levels after the prime, but after the boost the group primed with 200 µg of protein and boosted at day 23 had ∼1.5 fold higher HA-specific IgG levels as compared to the 125 µg primed and boosted group. As discussed later, this difference in immunological response does not conclusively imply a higher protective efficacy of the 200 µg dose. A 275 µg dosage, the highest dosage tested, also did not increase the primary immunogenic response compared to those observed for 125 and 200 µg dosages. Overall, the results demonstrate that HA_63–286_-RBD is immunogenic in the ferret model and is capable of triggering a specific IgG immune response.

**Table 2:** Results from an Influenza A H1N1 challenge experiment in ferrets.

Code	Dose I (µg)	Dose II (µg)	Symptom Indexes				Overall Sickness Index
			Δ T index (C)	Mucus	Sneezing index	Δ weigth index (C)	
VACCINATED							
1A	125a		**4.34**(‡)	1.50	1(†)	−1.16	1.07
1B	125a		−1.87	2.50	0	**−3.98**(‡)	1.18
2A	200	75	3.12(†)	**3.50** (‡)	0	−1.29	0.93
2B	200	75	2.19	1.50	0	−1.69	0.82
2C	200		1.59	1.50	0	−1.32	0.65
2D	200	75	2.75	**7.00**(‡)	0	**−3.42**(‡)	1.78(†)
2E	200		**5.44**(‡)	**3.50**(‡)	0	−**5.31**(‡)	**2.37**(‡)
2F	200		2.00	1.50	1(†)	−1.43	0.92
3A	275		−0.15	**4.00**(‡)	0	−1.77	0.79
3B	275		−0.63	**3.50**(‡)	**2**(‡)	−1.57	1.03
3C	275		−1.51	0.00	1(†)	−1.87	0.60
4A	125	75	2.15	2.00	**2**(‡)	−**3.39**(‡)	1.75(†)
4C	125	75	**5.69**(‡)	1.00	**2**(‡)	−2.73(†)	1.83(†)
4D	125		1.92	2.00	0	−0.52	0.48
4E	125		0.16	2.50	0	−**4.08**(‡)	1.41
4F	125		1.13	**3.00**(‡)	0	−2.41(†)	1.04
**sum**			28.34	40.50	9	−37.92	18.65
**average**			1.77	2.53	0.56	−2.37	1.17
**Std Dev.**			2.13	1.58	0.81	1.28	0.52
**CONTROLS (NON-VACCINATED)**							
C1	0	0	**8.04**(‡)	**6.00**(‡)	**3**(‡)	−2.43(†)	**2.50**(‡)
C2	0	0	**6.41**(‡)	**4.00**(‡)	**2**(‡)	**−3.45**(‡)	**2.32**(‡)
C3	0	0	**6.47**(‡)	**5.00**(‡)	**3**(‡)	−2.64(†)	**2.34**(‡)
C4	0	0	**6.31**(‡)	**3.00**(‡)	**2**(‡)	−**4.60**(‡)	**2.59**(‡)
C5	0	0	**8.48**(‡)	**3.00**(‡)	**3**(‡)	−**6.22**(‡)	**3.56**(‡)
**sum**			35.70	21.00	13.00	−19.34	13.31
**average**			7.14	4.20	2.60	−3.87	2.66
**Std Dev.**			0.93	1.17	0.49	1.40	0.46

Values for five symptom indicators and an overall sickness index are presented for each of 21 animals, distributed in 5 experimental groups, coded as 1,2,3,4,and C. Groups 1 to 4 were administered with different doses of HA_63–286_-RBD, in one or two immunizations (as indicated in column Dose I and Dose II). Group C was the negative control (non-vaccinated). Symbols were assigned based on magnitude deviation with respect to the average value for that particular indicator: no symbol for values lower than average; (†) for values around mean value; and (‡ and value in bold) for values significantly above the average.

Table 3 presents the results of a neutralization assay conducted on selected serum samples from immunized ferrets (ferret 1A, 2C, 2D, 2E, 4A, 4C, 4D, 4E) and a non-vaccinated control (ferret C3). The study was conducted such that all dosage groups were represented. All samples were tested at a dilution of 1∶40. This dilution was similar to the one used for the ELISA. Conventionally, it is accepted that a serum sample that neutralizes virus infection at a dilution of 1∶40 or higher will be at least partially protective [30]. All samples tested from vaccinated ferrets resulted in neutralization in at least 50% of the conducted replicates (see fourth column in Table 3). No obvious correlation can be established between neutralization potential and vaccine dosage, and even a single dosage of 125 µg was found to be capable of stimulating the production of neutralizing antibodies. Pre-immune samples and dilutions of 1∶160 of the vaccinated ferrets resulted non-neutralizing. Similarly, samples from a non-vaccinated control that became severely symptomatic after viral challenge (ferret C3) did not neutralized virus infection.

**Table 3:** Evaluation of neutralization of H1N1/2009 infection in MDCK cultures.

HA-RBD dose	Ferret identifier	Positive control^1^	Pre-immune serum^2^	Post-immune serum^3^^,^ 4	Observations
Single dose/125µg+adj	1A	**(+)**	(−)	**100% (+); 2/2**	Slightly symptomatic
Single dose/125 µg	4D	**(+)**	(−)	**75% (+); 3/4**	Slightly symptomatic
Single dose/125 µg	4E	**(+)**	(−)	**66% (+); 2/3**	Slightly symptomatic
Double dose/125 µg+75 µg	4A	**(+)**	(−)	**66% (+); 2/3**	Moderately symptomatic
Double dose/125 µg+75 µg	4C	**(+)**	(−)	**100% (+); 2/2**	Moderately symptomatic
Single dose/200 µg	2C	**(+)**	(−)	**100% (+); 2/2**	Slightly symptomatic
Single dose/200 µg	2E	**(+)**	(−)	**100% (+); 4/4**	Severely symptomatic
Double dose/200 µg+75 µg	2D	**(+)**	(−)	**50% (+); 1/2**	Moderately symptomatic
Non-vaccinated	C3	**(+)**	(−)	**0% (+); 0/2**	Severely symptomatic

Pre-immune serum samples from selected ferrets did not exhibit neutralization against H1N1/2009 infection. Thirty days post-immune diluted serum samples (1∶40) taken from vaccinated ferrets neutralized virus infection in MDCK cultures.

1 Human serum from a 30 days H1N1/2009 convalescent subject.

2 Serum sample taken at the moment of vaccination.

3 Serum sample taken 30 days after vaccination.

4 Percentage of assays that rendered neutralization at 1∶40 dilution.

### HA_63–286_-RBD protects ferrets against challenge by H1N1 influenza virus

Twenty-one ferrets (sixteen vaccinated and five non-vaccinated controls) were then challenged with influenza A/H1N1/2009 virus. The virus was cultured in MDCK cells in our laboratory using techniques reported elsewhere [6]. The identity of the virus was confirmed by a WHO-recommended real time RT-PCR protocol [27]. Briefly, ferrets were lightly anesthetized and a 200 µL aliquot of virus suspension from a cell culture supernatant with a virus titer of 10^5.83^ TCID50 mL^−1^ was administered intranasally at day 0 of the challenge experiment, 45 days after first vaccination. Body temperature (as measured by a microchip implanted in the animals four days before the challenge), weight, sneezing and the presence and appearance of mucus were monitored for one week after the challenge in all animals. The efficacy of infection was determined by verifying the presence of influenza A/H1N1/2009 virus through real time RT-PCR in pharyngeal samples taken at days 2 and 4 after challenge. All animals tested positive for H1N1 infection, with CT values in the range of 20 to 25 cycles at both days 2 and 4 post infection. These values are considered high in human samples, implying that infective concentrations of the virus were successfully delivered to each experimental subject.

Table 2 shows a summary of symptoms displayed by each ferret based on four commonly accepted indicators of disease [31], [32]. Each index was constructed such that its value range falls between 0 and 5. An overall sickness index (OSI) resulted from the weighted addition of all four individual symptom indexes, according to the expression:
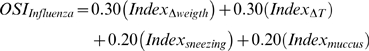
(1)


Weights were empirically assigned based on the relative significance of the symptom; increases in temperature and weight loss were assumed more relevant and objective than mucus quality and sneezing. Visual inspection of data revealed that lower intensity influenza symptoms were observed in vaccinated ferrets. The overall sickness index (OSI) provides a better estimate of the severity of symptoms in vaccinated and non-vaccinated animals. The average OSI for all ferrets was 1.6 units, and ferrets with OSI values lower than this value were rated as slightly symptomatic. Subjects with OSI values between 1.8 and 2.0 were rated as moderately symptomatic, and animals with OSI values above 2.3 units were rated as severely symptomatic. From the vaccinated population, only 6.25% (1/16) of the animals were identified as severely symptomatic and 18.75% (3/16) exhibited moderate influenza symptoms. In contrast, 100% of non-vaccinated ferrets (5 out of 5 subjects) exhibited severe influenza symptoms. Ferret 2E, the only vaccinated ferret that displayed severe influenza symptoms, received a single but relatively high dose of protein HA_63–286_-RBD that resulted in high titers of specific antibodies (as measured by ELISA [28]). In addition, 1∶40 dilutions of its serum samples were capable of neutralizing virus infection in MCDK cultures (Table 3).

**Figure 7:**
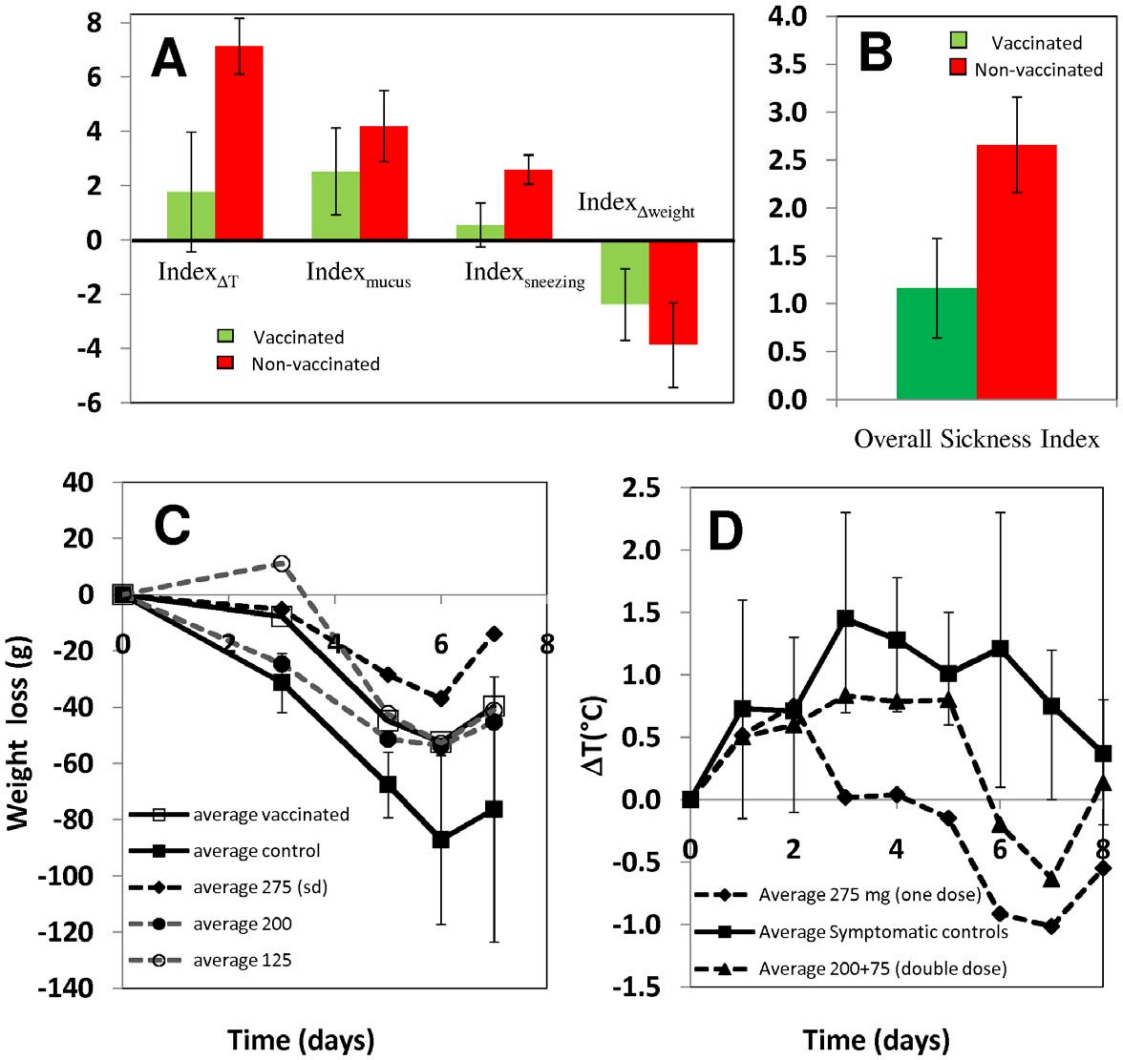
Protective effect of HA_63–286_-RBD in ferrets challenged with influenza A/H1N1/2009 virus. (A) Averages from four different symptom indexes are compared for vaccinated (green bars) and non-vaccinated ferrets (red bars). Error bars represent one standard deviation. (B) Averages of the overall sickness index (as defined in text) are compared for vaccinated (green bars) and non-vaccinated ferrets (red bars). Error bars represent one standard deviation. (C) Evolution of body weight loss in ferrets challenged intra-nasally with infective dosages of influenza A/H1N1/2009 virus (at day 0). Averages of daily Δweight (Weight_dayx_–Weight_basal_) for five non-vaccinated controls (▪), five ferrets administered with a single dose of 125µg of HA_63–286_-RBD (○); three ferrets administered with a single dose of 200 µg of HA_63–286_-RBD (•); three ferrets administered with a single dose of 275 µg of HA_63–286_-RBD (♦); and all sixteen vaccinated ferrets (□) are compared. Error bars represent one standard deviation. (D) Evolution of body temperature in ferrets challenged intra-nasally with infective dosages of Influenza A/H1N1/2009 virus (at day 0). Averages of daily ΔT (T_dayx_–T_basal_) for five non-vaccinated controls (▪), five ferrets administered with a single dose of 275 µg of HA_63–286_-RBD in a double dose (▴), and 275 µg of HA_63–286_-RBD in a single dose are compared (♦). Error bars represent one standard deviation.

OSI values did not exhibit a normal distribution, and a Mann-Whitney test based on median comparisons was therefore conducted. OSI was significantly lower in the vaccinated than in the non-vaccinated group (P_value_<0.001). Comparatively, all individual sickness indicators were significantly higher in non-vaccinated than in vaccinated ferrets (Figure 7a,b). Average Index_ΔT_ was significantly higher in non-vaccinated ferrets according to a T test (P_value_<0.001). In non-vaccinated ferrets the average maximum weight loss was significantly more severe (T test; P_value_ = 0.056) and the average Index_mucus_ was also significantly higher (T test; P_value_<0.023). Values for Index_sneezing_ did not exhibit a normal distribution and were analyzed using a sign test based on a comparison of medians. Index_sneezing_ was significantly higher in non-vaccinated ferrets (P_value_ = 0.001). Significant differences in temperature excursion profiles and weight loss profiles were observed in vaccinated and non-vaccinated ferrets (Figure 7c,d). At the moment of sacrifice (three weeks after challenge), all non-vaccinated animals tested positive to the H1N1/2009 virus by PCR, while only 40% of immunized animals did.

## Discussion

### Is glycosylation crucial for efficacy?

Our results suggest that the non-glycosylated, refolded HA1 receptor-binding domain produced in *E. coli* is immunogenic and elicits protective immunity *in vivo*. The HA_63–286_-RBD protein triggered specific immunogenicity (as determined by ELISA [28]), caused the production of neutralizing antibodies, and induced protection against infection in ferrets. To our knowledge, this is the first report of a recombinant vaccine against influenza A/H1N1/2009 produced in bacteria and successfully tested in the ferret model. HA_63–286_-RBD is also the smallest HA fragment successfully tested as an influenza vaccine in animal models. Previous reports of protective HA subunits refer to fragments of more than 328 [10], [12] and 720 amino acid residues [11].

Although the relationship between HA glycosylation, immunogenicity, and protective capacity of anti-influenza vaccines has not been clearly established [5], [19], it is generally assumed that “proper” glycosylation is a mandatory requirement for adequate function. Our findings challenge this concept and re-open questions about the underlying mechanisms of immunogenicity. Four antigenic sites, Sa, Sb, Ca (composed of the Ca1 and Ca2 subunits) and Cb, have been conclusively identified in the globular region of hemaglutinnin from influenza A H1N1 viruses [22], [33]–[39]. Although some of these are in the vicinity of glycosylation sites, none are glycosylated [17], [22], [36]. Moreover, some native HA proteins have fewer glycosylation sites than others. For example, the HA protein from H1N1/2009 or H5N1 viruses have fewer glycosylation sites than the HA proteins of currently circulating human H3N2 viruses. In particular, a recently published structural analysis of the HA protein of the influenza A/H1N1/2009 virus [40] shows that its globular region is only glycosylated at one site (only one Asp at the protein surface). This glycosylation site does not interfere with any of the antigenic sites anticipated by simulations. The same computational study [22] predicts that only one of the five antigenic sites of the HA protein of the H1N1/2009 could become glycosylated by a single nucleotide mutation. In addition, all typical antigenic sites in other HA molecules have been reported to be accessible to specific antibodies [34]–[38]. Early work by Skehel *et al.*, suggested that glycosylation could even interfere with proper antibody recognition [41]. It was recently demonstrated that HA molecules with poor or truncated glycan structures have similar secondary structures, and exhibit higher binding affinity for cellular receptors and anti-HA antibodies than do the fully glycosylated forms [19]. Significantly, superior protective performance was observed with HA with truncated glycans [19]. Antibodies from sera of animals administered with a monoglycosylated HA molecule exhibited higher binding affinities for native HAs and stronger neutralization of the virus. Notably, in lethal challenge experiments in mice using an H5N1 virus, an HA version with truncated glycosylation exhibited higher protective efficacy.

### Practical relevance

The efficacy of the recombinant protein presented here has yet to be compared to currently available Influenza A/H1N1 vaccines produced in embryonated chicken eggs. Thus far, we have only used antigen concentrations of 125–275 µg/dose in our experiments with ferrets. These dosages are one order of magnitude higher than those previously used in similar experiments with adjuvanted influenza virus vaccines [42], [43] (1.5 to 15 µg/dose). Our results indicate that a single dose of 125 µg of HA_63–286_-RBD is safe in ferrets and could provide adequate protection (above 90%) with no need of adjuvant. This has practical importance because adjuvant use has not been approved for human use in many countries. A recent report documents that rabbit serum antibodies raised against a full-length, glycosylated HA-H5N1 produced in a baculovirus-insect cell system (insect cells produce “trimmed” glycosylation compared to egg/mammalian cell systems) exhibited four times higher viral neutralizing titer than serum antibodies against an HA1-H5N1 produced in *E. coli* (12). As the *E. coli* construct was not shown to fold into its proper antigenic structure, this difference in immunogenicity cannot be solely attributed to differences in glycosylation. However, even accepting that the potency of the non-glycosylated recombinant HA_63–286_-RBD described here could be an order of magnitude lower than that of current influenza virus vaccines, a bacterial system will produce at least 1000 more doses per unit of time per unit of volume than current egg-based systems, and at least 100 more doses than other technologies currently being explored. Typical productivity of egg-based technologies can be calculated at ≈30–40 µg L^−1^ h^−1^. According to average final concentrations and yields reported in literature, the expected yields for emerging technologies such as viral culture in insect or mammalian cells are ≈1000 µg L^−1^ h^−1^ [3], [11], [44], [45]. In contrast, the expected productivities for recombinant expression of a single antigenic protein in bacterial cultures are on the order of ≈210,000 µg L^−1^ h^1^. Considering levels of expression and recovery yields known for production of complex recombinant proteins in *E. coli* cultures, i.e. (HA)_final_ = 3.0 g/L after 24 hours of process [45] and recovery yields = 0.4 [46], [47], the technology presented here would allow the production of 2.2 million doses of influenza vaccine in a conventional 1,000 L bioreactor (pilot plant scale) per day, even if we considered a dose of 450 µg/person which represents one order of magnitude higher than conventional viral HA equivalent.

Influenza vaccines based on HA fragments produced in *E. coli* could be the next generation of commercial influenza vaccines. Recently, other groups have also reported experimental evidence for the potential of *E. coli* platforms for influenza vaccine production [3], [10]–[12]. As an illustration, the activity of the complete HA1 domain of the hemagglutinin of the influenza H5N1 virus produced in *E. coli* cultures, exclusively measured in terms of specific recognition from infected rat serum antibodies, was found to be strongly dependent on the refolding method [10]. Song *et al.* recently proposed a bacterial expression vaccine platform as a cost and time effective solution for pandemic and seasonal influenza outbreaks [11]. The authors demonstrated that recombinant fusion proteins consisting of HA1 subunits linked to the Toll-like receptor 5 (TLR5) ligand (flagellin) produced in *E. coli* displayed strong immunogenic and protective activity in rodents [11], although the protective activity of the HA1 subunits themselves was not studied. Biesova *et al.* reported [12] that an adjuvanted formulation based on an HA-H5N1 recombinant fragment of 60 kDa produced in *E. coli* showed antigenic and protective activity in mice. The design and production strategies that we have developed represent a major advance in this approach and could be a general low cost and high volume platform for the rapid and copious production of pandemic and seasonal influenza vaccines.

## Materials and Methods

### Genetic construction

We expressed a 25 kDa fragment of the globular region of the hemagglutinin of the influenza A/H1N1/2009 virus, from residues 63 to 286 (v.gr. GenBank accession No. ACQ99608), in three strains of *E. coli*: Rosetta-gami™ (DE3) pLysS from Novagen® (EMD4 Biosciences, NJ), and BL21 (DE3) pLysS variants C41 and C43 obtained from Lucigen® Corporation (Middleton, WI). The protein is referred to here as HA_63–286_-RBD. A sequence coding for a series of six histidines was added at the N-terminus of the protein followed by an enterokinase recognition site to facilitate removal of the 6His tag (Figure 1). The corresponding DNA sequence was obtained by back translation of the open reading frame and was optimized for *E. coli* expression. This gene was synthesized at DNA2.0 (Menlo Park, CA), and cloned in a pJexpress404 vector.

### Production in bioreactors

*E. coli* culture experiments in LB medium were conducted in instrumented bioreactors at scales of 250 mL and 5 L, under culture conditions of 37°C, pH 7.0, and 20% dissolved oxygen. Protein production was induced during early exponential growth phase, once optical density (as measured at 580 nm) reached values between 0.6 and 0.8 absorbance units, by the addition of 0.4 to 1 mM IPTG (isopropyl-tiogalactoside or 1-metil-etil 1-tio- β-D-galactopiranoside). After induction, culture was extended for eight to ten hours at 30°C.

### Recovery, purification and refolding

After cultivation, biomass was centrifuged at 3000g for 10 minutes. 20 mL of TALON® xTtractor Buffer (Clontech Laboratories, Inc.) were added per gram of wet cellular pellet to disrupt cell membranes and extract the inclusion bodies. A concentrated solution of type I DNAses and Lysozyme 1X, was added to further degrade cell membranes and degrade DNA, consequently decreasing the viscosity of the solution and facilitating further processing. The resulting solution was centrifuged at 12,000g for 30 minutes at 4°C. A series of consecutive washing steps using PBS buffer rendered a precipitate containing the protein of interest, in its insoluble form, with purity higher than 90%. The recombinant protein was dissolved in an 8 M urea, and the protein solution was loaded onto chromatography columns containing 2 mL of TALON® Metal Affinity Resin (Clontech Laboratories, Mountain View, CA) containing Co^2+^ ions and equilibrated at pH 8. While still attached to the resin via its 6His tag, HA_63–286_-RBD was treated with successive PBS or 400 mM arginine washes at pH 7 or 8 respectively to promote refolding. Protein was eluted using 150 mM imidazole at pH 7. The resulting protein solution was dialyzed to remove imidazole and quantified by microelectrophoresis using an Experion® platform from Bio-rad (Hercules, CA). For these studies, the 6His tag and EK-sequence was not removed.

### 3D structure modeling

Three dimensional structures were predicted using Swiss-model. This program uses an algorithm that finds the most thermodynamically stable 3D structure by minimization of the free Gibbs energy of a preliminary inferred structure. To calculate this preliminary structure, the algorithm requires as inputs (a) the amino acid sequence of protein HA_63–286_-RBD, and (b) and the amino acid sequence and crystal structure reported for a structurally similar protein. Here, the full length HA of the Influenza A H1N1/1918 virus [23] was taken as template for the estimation of the most probable structure of protein HA_63–286_-RBD. Visualization and highlighting of immunogenic sites was done using UCSF-Chimera. The structure of the antigenic epitopes of the HA of the influenza A H1N1/CA2009 virus was taken from Igarashi *et al.* [22].

### Analytical Ultracentrifugation

Experiments were carried out in a ProteomeLab XL-I analytical ultracentrifuge with a four-hole rotor (Beckman An-60Ti) and cells containing sapphire or quartz windows and charcoal-filled Epon double-sector centre pieces (Beckman Coulter, Fullerton, CA). The density and viscosity of the ultracentrifugation buffer, 10 mM Tris pH 8.0, 100 mM NaCl at 4 and 20°C were calculated from its composition and the partial specific volume at 4 and 20°C and the molecular weight of the protein was calculated based on its amino acid composition using the software SEDNTERP [48]. Buffer from the size-exclusion column was used as the ultracentrifugation buffer and optical reference. For the sedimentation velocity experiment the loading volume of 400 µl was identical for the reference and sample chambers of the double-sector centrepiece. Fringe displacement data at time intervals of 1.0 min were collected with the Rayleigh interference system for 10 hours at a rotor speed of 60,000 rpm and analysed with SEDFIT software (www.analyticalultracentrifugation.com) using the model for continuous sedimentation coefficient distribution *c(s)* with deconvolution of diffusional effects [49], [50]. The sedimentation coefficient distribution *c(s)* was calculated with maximum entropy regularization at a confidence level of p = 0.7 and at a resolution of sedimentation coefficients of n = 100. The positions of the meniscus and bottom, as well as time-invariant and radial noises, were fitted. Sedimentation equilibrium was attained at 24 h at a rotor temperature of 4°C at increasing speeds of 20, 30 & 38 k rpm [51]. Protein at concentrations of between 5 and 17 µM (120 µL) were loaded into double-sector centrepieces and absorbance distributions recorded at 280 and 250 nm in 0.001 cm radial intervals with 20 replicates for each point. Global least squares modelling were performed at multiple rotor speeds with the software SEDPHAT using a reversible monomer-dimer self-association model as well as the single species model [51].

### ELISA protocol

Specific binding to antibodies from serum samples of Influenza A/H1N1/2009 convalescent patients was determined by a specific ELISA protocol using protein HA_63–286_-RBD as antigen ([28]; Figure S1). All patients provided written informed consent for the collection of samples and subsequent analysis at the moment that the blood sample was taken. This study was conducted according to the principles expressed in the Declaration of Helsinki. The study was approved by the Institutional Review Board of the School of Biotechnology and Health at Tecnológico de Monterrey at Monterrey, México. Anti-histidine antibodies were fixed to the surface of 96 well immunoassay microplates. A commercial solution was used to block surface spaces within the plate. HA_63–286_-RBD was added to the micro-wells, and non-attached excess was removed by successive washes with PBS. Serum samples to be assayed (1∶50 dilution in PBS) were added to each well to test for specific bio-recognition.

Serum samples were incubated at room temperature for one hour. After incubation, wells were washed repeatedly. To reveal the amount of antibody specifically bound in each well, a volume of 100 µL/well of an anti-human IgG antibody solution (1∶30000 dilution in PBS-Tween 0.05%) marked with horse radish peroxidase (Pierce®, USA) was used. After incubating for one hour at room temperature and washing repeatedly, a 100 µL volume of substrate solution (1-Step Ultra TMB-ELISA; Lot. 34028, Pierce®) was added to each well. After incubation for 15±5 min at room temperature in darkness, the enzymatic reaction was stopped by addition of 50 µL/well of a 1M H_2_SO_4_. Color produced by the enzymatic reaction (from colorless to yellow) was evaluated by absorbance at 450nm in a Biotek® microplate reader, USA [28].

### Immunology studies in ferrets

Experiments with animal models were conducted in accordance with international, national (NOM-062-ZOO-1999) and institutional guidelines. In particular, the immunization and challenge experiments documented in this study were submitted to and approved by the Institutional Committee for humanitarian animal use and care of the School of Biotechnology and Health of the Tecnológico de Monterrey (Protocol #11 H1N1 CB, approved on 11/09/2009; and Protocol #16 H1N1 CB, approved on 11/11/2009). All recommendations of the committee were followed to minimize animal suffering or unnecessary manipulation.

The immunogenic potential of HA_63–286_-RBD was evaluated in experiments with 16 ferrets (*Mustela putorius* furo: age approximately 8 months, bodyweight 0.8–1.5 kg). Sixteen ferrets were administered intramuscularly with different concentrations of HA_63–286_-RBD: 275, 200, and 125 µg of protein without adjuvant and 125 µg of protein with adjuvant MF59C.1, Novartis® (containing 9.3 mg of esqualene). Selected animals were administered with a second dose of 75 µg of protein (without adjuvant) fifteen days after the first dose was applied (Table 2). Five additional animals were used as non-vaccinated controls (C1–C5). Blood samples were taken from each vaccinated animal at day 3, 7, 12, 16, 20, 23, 27, 30 and 34. Serum was isolated and analyzed for specific antibodies using the ELISA technique previously described, with the only exception of using a peroxidated anti-ferret antibody instead of an anti-human one.

### Neutralization experiments

Neutralization assays were conducted with serum samples from selected vaccinated ferrets (ferret 1A, 2C, 4B, 4C, 4D, 4E, 4F and a non-vaccinated control (ferret C3). MCDK cells were cultured to confluency in 96 well micro-plates at 30°C in DMEM culture medium. To infect cells, a stock of H1N1/2009 viral suspension with a 10^2^ TCID50/mL titer was used. Cytopathic effect was observed when 100 µL of a 1∶1 dilution of this viral suspension in PBS was administered to confluent cell cultures (for a virus final titer of 5^2^ TCID50/mL). Similarly, to test neutralization of each serum sample, 50 µL of the undiluted viral stock suspension and 50 µL of a 1∶20 dilution of the serum sample in PBS were administered to confluent cell cultures (for a final virus titer of 5^2^ TCID50/mL and a final serum dilution of 1∶40) in each well. Serum samples were diluted 1∶160 in K-PBS to serve the purpose of negative controls. After an incubation period of 1 hour at 30°C, the viral suspension and serum solution was washed twice from each well with culture media and 200 µL of medium supplemented with 0.20% of BSA and 2 µg/mL of TPCK trypsin. At least two replicates for each serum sample were conducted.

### Protective studies in ferrets

The protective potential of HA_63–286_-RBD was evaluated in experiments with 21 ferrets, 16 vaccinated animals and 5 controls. Vaccinated ferrets were challenged intra-nasally 45 days after first vaccination with 200 µL of Influenza A/H1N1/2009 virus suspension from a MDCK cell culture supernatant with a virus titer of 10^5.83^ TCID50 mL^−1^. The influenza A/H1N1/2009 virus strain used was isolated from an infected subject and was kindly donated by INDRE (Instituto Nacional de Referencia Epidemiológica, México).

Four symptoms of influenza infection were monitored in experimental animals during seven days after intra-nasal viral challenge with 200 µL of virus suspension from a MDCK cell culture supernatant with a virus titer of 10^5.83^ TCID50 mL^−1^. For each symptom, an indicator was defined. The temperature excursion index (Index_ΔT_) equals the sum of ΔT values (T_day x_–T_day 0_) from day 0 to day 7 of the experiment. T_day x_ was determined as the average of three scanning lectures of the micro-ship implanted in each animal, always taken at the same time of the day and at least 15 minutes after the animals were fully awaken. The sneezing index (Index_sneezing_) was built as the sum of days at which the animal exhibited frequent events of consecutive sneezing. Sneezing was exclusively monitored at days 3, 5, and 6. Mucus quality was graded in a scale of 0 to 3 (0- no mucus is evident; 1- transparent mucus is present; 2- colored mucus present; 3- abundant and colored mucus is present). The mucus index (Index_mucus_) was calculated as the sum of daily values, from 0 to 3, registered for mucus quality at days 3, 5, and 6. Weight loss index (Index_Δweight_) was calculated as the maximum loss in weight (grams/25 grams) with respect to body weight registered at the beginning of the challenge. In all cases, maximum weight loss was observed at day 6 or 7 of the challenge.

## Supporting Information

Figure S1 Schematic representation of an immunoassay used to validate the preferential biological affinity of the recombinant protein for antibodies present in serum of patients infected with influenza A H1N1/2009. (A) Anti-histidine antibodies were fixed to the surface of 96 well immunoassay microplates. (B) After blocking with a commercial agent, a solution of protein HA_63–286_-RBD was added to each well. (C) In comparative experiments, serum samples (1∶50 dilution) from positive and negative volunteers were added; left panel illustrates a scenario with a higher concentration of specific influenza antibodies. (D) Addition of a peroxidated anti-IgG human antibody to specifically bind the retained serum antibodies. The addition of peroxidase substrate enables the enzymatic peroxidation with an associated proportional development of colour.
(1.82 MB TIF)
