# Supplementary figures and images for: An Influenza A/H1N1/2009 Hemagglutinin Vaccine Produced in Escherichia coli
Source: PLoS One. 2010 Jul 22;5(7):e11694. doi: 10.1371/journal.pone.0011694 (PMC2908544; doi:10.1371/journal.pone.0011694)

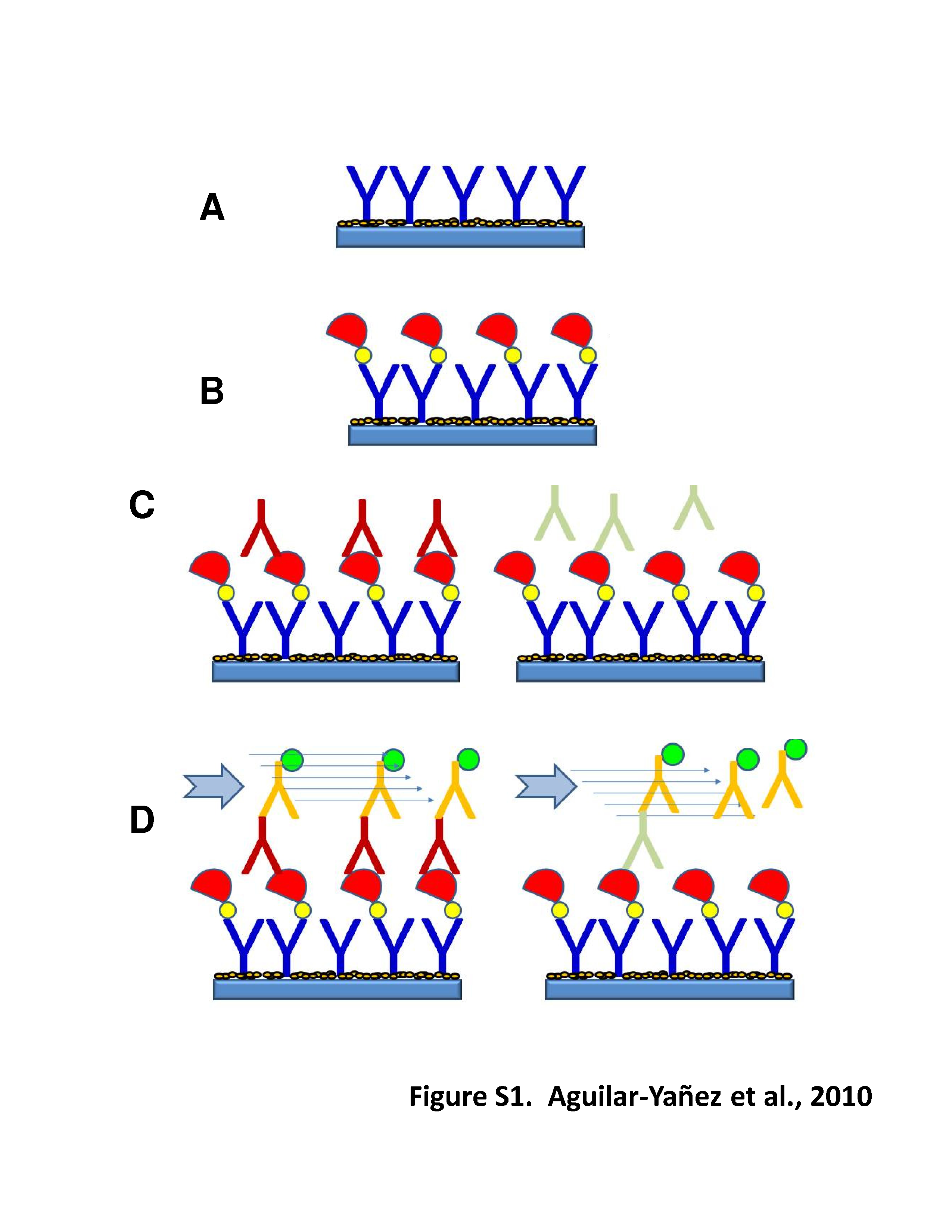

Supplement: Figure S1 — Schematic representation of an immunoassay used to validate the preferential biological affinity of the recombinant protein for antibodies present in serum of patients infected with influenza A H1N1/2009. (A) Anti-histidine antibodies were fixed to the surface of 96 well immunoassay microplates. (B) After blocking with a commercial agent, a solution of protein HA63–286-RBD was added to each well. (C) In comparative experiments, serum samples (1∶50 dilution) from positive and negative volunteers were added; left panel illustrates a scenario with a higher concentration of specific influenza antibodies. (D) Addition of a peroxidated anti-IgG human antibody to specifically bind the retained serum antibodies. The addition of peroxidase substrate enables the enzymatic peroxidation with an associated proportional development of colour. (1.82 MB TIF) [file pone.0011694.s001.tif]
